# Murder on the Ovarian Express: A Tale of Non-Autonomous Cell Death in the *Drosophila* Ovary

**DOI:** 10.3390/cells10061454

**Published:** 2021-06-10

**Authors:** Diane Patricia Vig Lebo, Kimberly McCall

**Affiliations:** Department of Biology, 5 Cummington Mall, Boston University, Boston, MA 02215, USA; dlebo@bu.edu

**Keywords:** *Drosophila*, ovary, oogenesis, cell corpse clearance, phagocytosis, efferocytosis, phagoptosis

## Abstract

Throughout oogenesis, *Drosophila* egg chambers traverse the fine line between survival and death. After surviving the ten early and middle stages of oogenesis, egg chambers drastically change their size and structure to produce fully developed oocytes. The development of an oocyte comes at a cost, the price is the lives of the oocyte’s 15 siblings, the nurse cells. These nurse cells do not die of their own accord. Their death is dependent upon their neighbors—the stretch follicle cells. Stretch follicle cells are nonprofessional phagocytes that spend the final stages of oogenesis surrounding the nurse cells and subsequently forcing the nurse cells to give up everything for the sake of the oocyte. In this review, we provide an overview of cell death in the ovary, with a focus on recent findings concerning this phagocyte-dependent non-autonomous cell death.

## 1. Introduction

More than 300 billion cells die in a human body every day [1,2]. Although this number may seem daunting, this is a typical day for a healthy human adult. Cells, such as erythrocytes and short-lived gut epithelial cells, become worn out over time and need to be replaced to maintain homeostasis [1,2]. These death events, however, are not random, uncontrolled phenomena.

Scientists and physicians have been studying cell death for over 200 years [3,4]. In that time, they have identified several types of cell death, but it was not until 1964 that the term programmed cell death was first published [5]. This form of cell death, which was later renamed regulated cell death (RCD), was defined as internally-controlled cell death [6]. Nearly ten years later, the first type of RCD was defined and given the name apoptosis [7].

In apoptotic cell death, the nucleus condenses and fragments, followed by the release of membrane-bound cellular fragments which are then engulfed by nearby phagocytes [7]. Over the past 50 years, our knowledge of apoptosis has grown from a morphological description into two separate complex molecular mechanisms, the intrinsic and extrinsic apoptotic pathways, with executioner caspases playing central roles in both [6,8,9]. As our ability to study processes at a molecular level has expanded, over a dozen other forms of cell death have also been identified [6,8]. Originally, each new form of cell death was placed into one of four categories—apoptotic, autophagic, necrotic, and atypical—based on their morphology [8]. The growing diversity of RCD, however, has necessitated that the different forms of cell death be defined by their molecular characteristics [6].

Although each of these cell death paradigms differs greatly from the next, they all produce the same end result: dead or dying cells. These corpses are potentially dangerous to their surrounding cells as membrane integrity is no longer being actively maintained [10,11,12,13]. Once the intracellular contents, such as ATP and uric acid, are released into the extracellular milieu, they may act as damage associated molecular patterns (DAMPs). DAMPs bind to receptors on neighboring cells thus triggering a pro-inflammatory response [10,11,12,13]. If left untempered, the pro-inflammatory response can lead to further cell death and autoimmune disease [14,15,16,17].

To avoid the release of DAMPs, multicellular organisms possess phagocytic cells that clear away dying cells before their membranes permeabilize [10,12]. Phagocytes have been separated into two categories based on their main functions [2,16,18,19]. Macrophages and dendritic cells, whose main purpose is to clear away dying cells and debris, are called professional phagocytes. While professional phagocytes are efficient at clearance and even participate in the innate immune response to pathogens, they cannot enter certain tissues [16]. Such immunoprivileged tissues, which include the retina and testis, are therefore reliant on their resident nonprofessional phagocytes [16,20,21]. Nonprofessional phagocytes are cells that have a primary role other than clearance. When nonprofessional phagocytes encounter a cell corpse, their phagocytic machinery is engaged to clear it away. Many types of epithelial cells and fibroblasts can behave as nonprofessional phagocytes [2,16,19].

In recent years, the relationship between cell death and corpse clearance has become more intimate as a new form of cell death known as phagoptosis was identified [22,23,24,25,26,27]. Phagoptosis has been loosely described as a form of RCD that requires the phagocytic machinery of another cell. Just as apoptosis and other forms of cell death have expanded and diversified over the years, so too has phagoptosis. Phagocytes have been shown to promote cell death to different extents; for instance, nonprofessional phagocytes are required for the assisted suicide of vas deferens progenitor cells in *C. elegans* and mammalian microglia have been shown to murder healthy PC12 neuronal precursors [23,28]. *Drosophila melanogaster* has proven to be a valuable model for studying phagoptosis as several examples of phagocyte-dependent cell death have been reported, with two forms identified in the ovary [27,29,30,31]. 

The ovary of *Drosophila* is an excellent tissue for studying cell death and corpse clearance as several death events take place throughout oogenesis [32,33,34,35,36,37,38]. Some of these cell death events allow *Drosophila* to regulate their energy and egg output in order to give the next generation its best chance for survival [19,32,33,35,36,37,39]. During developmental death at the end of oogenesis, 15 nurse cells (NCs) are eliminated to produce a fully developed oocyte [32,33,40,41]. In this review, we will describe different forms of cell death in the ovary and highlight recent findings that have demonstrated that this NC death and clearance is dependent on the surrounding follicle cells.

## 2. *Drosophila melanogaster*, a Powerful System for Cell Biology Research

Though many people see fruit flies as irritating, they are marvels for geneticists and cellular biologists [42]. As a long standing model organism, there are many advantages to using *Drosophila melanogaster* for research, as they are small, inexpensive, easy to maintain, and quick to reproduce. Moreover, a myriad of genetic tools have been developed for working with *Drosophila*, including chromosome balancers, the GAL4-UAS and Q systems, RNAi, and more recently CRISPR/Cas9. The *Drosophila* genome is relatively simple, sequenced, and copiously annotated for quick study. Finally, as many as 70% of human disease genes are evolutionarily conserved in the fly, thus we can learn more about ourselves by examining our Dipteran neighbors [33,42,43,44,45,46]. 

### 2.1. The Ovary, a Structurally Simple Tissue 

**Figure 1:**
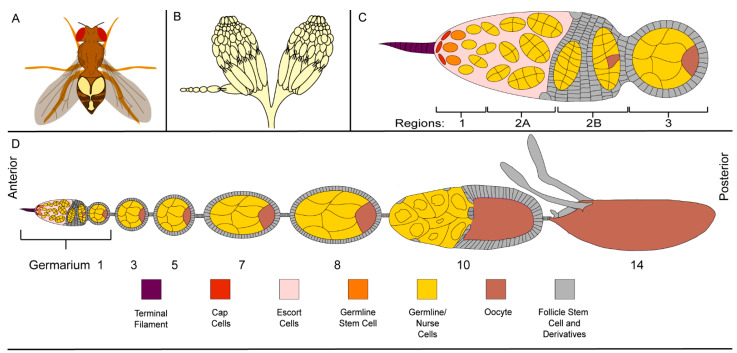
Structure of the *Drosophila* ovary (**A**) The relative size and position of ovaries within a *Drosophila* female. (**B**) A schematic of *Drosophila* ovaries showing an ovariole and its egg chambers. (**C**) The structure of a germarium with each of the germline cells, stem cells and niche in color and somatic cells in grey (see color scheme in Figure 1D). The terminal filament, cap cells, and escort cells found in region 1 act as the germline stem cell niche. Each egg chamber in the ovariole is derived, in part, from one of the germline stem cells. (**D**) A detailed schematic of an ovariole with the germline cells, stem cells, and niche in color and somatic cells in grey. An ovariole is composed of several egg chambers at different stages of development.

Although *Drosophila* are small animals, there are several accessible tissues for studying cell death and clearance including the brain, fat body, and ovaries [47,48]. The ovaries are particularly convenient as they are comparatively large tissues filling at least 50% of the fly’s abdomen, making them easy to visualize and dissect (Figure 1A) [49]. Using a simple light microscope, one can see that the general structure of the ovary is comprised of 15–20 strings of oblong units (Figure 1B) [40]. The strings are referred to as ovarioles and the oblong units are a series of progressively developing egg chambers. Egg chambers are produced from stem cells at the anterior-most region of the ovariole, in a structure called the germarium (Figure 1C,D). The most developed egg chambers are found at the posterior end of the ovariole, near the oviduct, through which their oocytes will be oviposited (Figure 1D) [40,50].

### 2.2. Germline Development

The germline cells become specified in the germarium which is composed of four regions—1, 2A, 2B, and 3 (Figure 1C,D) [51,52]. In region 1, each germline stem cell undergoes asymmetrical division to produce a cell, called the cystoblast, that will form the germline of an individual egg chamber [41,53,54,55]. The cystoblast undergoes four rounds of mitosis and incomplete cytokinesis to form a syncytium of fifteen nurse cells (NCs) and a single oocyte connected by ring canals [40,41,52]. Although they are produced from the same cell, NCs are easily distinguishable from the oocyte. 

**Figure 2:**
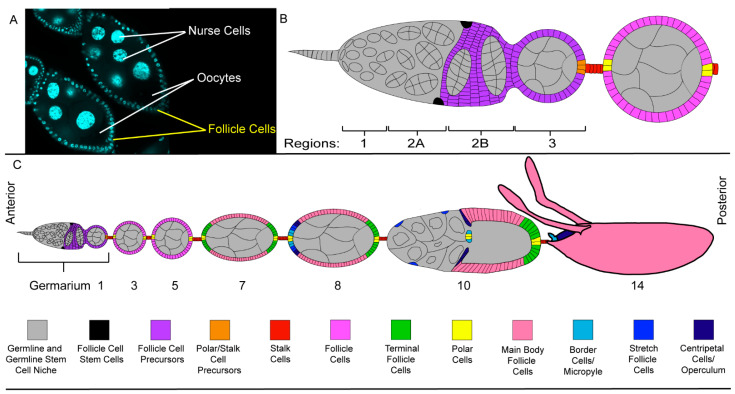
Somatic Cells of the *Drosophila* ovary (**A**) DAPI-stained egg chambers containing 15 NCs and a single oocyte. This syncytium of 16 cells is surrounded by a layer of smaller epithelial FCs. (**B**) A detailed schematic of an ovariole with the somatic cells in color and the germline cells, stem cells, and niche in grey (see color scheme in Figure 2C). The FCs are produced by the follicle cell stem cells found in region 2A of the germarium. (**C**) A detailed schematic of an ovariole with the somatic cells in color and the germline cells, stem cells, and niche in grey.

During early oogenesis, the NCs undergo several rounds of endocycling in which they replicate their DNA, but do not divide [56]. These 10–12 rounds of endocycling produce large NCs with large polyploid nuclei, while the oocyte nucleus remains small and dormant [56]. Thus, with a simple DNA stain, the oocyte can easily be identified in the cyst predominantly made up of NCs (Figure 2A). The oocyte and NCs, however, are not the only cells embarking on the journey of oogenesis.

### 2.3. The Follicle Cell Layer

After the germline syncytium is produced, it enters region 2A, where it encounters somatic follicle cell stem cells (Figure 2B) [53,57,58,59]. The somatic follicle cell stem cells proceed through their own round of asymmetric division to produce the follicle cell (FC) layer that will surround the germline in region 2B. As they pass through the rest of the germarium, the NCs, oocyte, and FCs organize themselves such that the oocyte is the posterior-most germline cell adjacent to the 15 NCs with the entire germline encapsulated by a single layer of FCs (Figure 2B) [40,58,59]. Together, this arrangement makes up an egg chamber.

Just as the NCs are easily distinguishable from the oocyte, the FC layer is easily distinguishable from its germline neighbors. While genetic markers can be used to differentiate the germline and FCs, respectively, a microscope and a nuclear stain will suffice (Figure 2A) [50]. Once stained with a nuclear dye, the FCs are easily visualized around the periphery of each egg chamber. The FCs are much smaller than the germline and the FC nuclei are much smaller than those of the NCs. 

As the egg chamber forms, the FCs begin to differentiate into three cell types—the epithelial FCs which surround the germline, the stalk cells which separate the egg chambers, and the polar cells which regulate the orientation of the oocyte and FC behavior (Figure 2B,C) [58,59]. During the early stages of oogenesis, as the oocyte grows the FCs divide until they reach a population of approximately 900 cells [58,59,60]. 

As the egg chamber continues to develop, signaling from the polar cells defines terminal regions at either end of the egg chamber (Figure 2C). FCs that do not receive polar cell signaling and make up a majority of the FC population are called main body FCs. Further signaling from the anterior polar cells differentiates the anterior terminal FCs into three separate groups—border cells which transport the polar cells to the oocyte, stretch FCs which are squamous cells that surround the NCs and act as nonprofessional phagocytes, and centripetal cells which migrate along the border between the NCs and oocyte where they will form structures for the fully developed oocyte [58,59,61,62]. 

Egg chambers traverse 14 stages of development to produce a fully formed oocyte which is structurally quite different from the original egg chamber (Figure 1D and Figure 2C) [40]. In early oogenesis, the NCs and oocyte are approximately the same size and are surrounded by a cuboidal FC layer. During mid-oogenesis, the egg chamber undergoes a remodeling event, where the oocyte increases in size compared to the NCs, while the main body FCs become columnar in shape [43,63]. As the columnar shape takes up less area and the oocyte is growing, there is a limited number of main body FCs to surround the oocyte. The remaining FCs become stretch FCs, squamous epithelial cells that surround the NC region of the oocyte. Once the remodeling event is complete, only 50 stretch FCs cover the NCs. As the newly remodeled egg chamber completes late oogenesis, just one cell remains, the oocyte [37,40,63,64].

## 3. Development, Death, and Nonprofessional Phagocytes during Early and Mid Oogenesis

**Figure 3:**
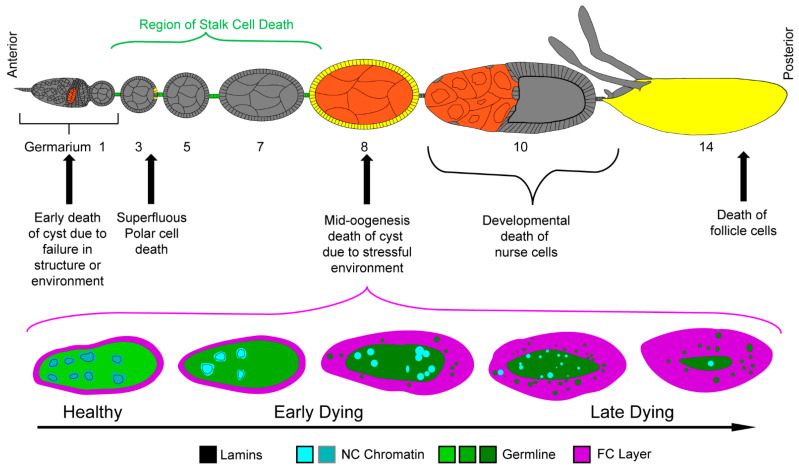
Cell death in oogenesis. Top—Schematic of ovariole with germline cell death events highlighted in orange and somatic cell death events highlighted in yellow and green. Bottom—Phases of cell death in egg chambers during mid-oogenesis are illustrated. Healthy egg chambers contain NCs with dispersed chromatin and intact nuclear lamina. The FC layer (magenta) surrounding the syncytium is thin, just 1 cell thick. As the germline dies, the chromatin condenses (as shown by the increasing brightness of the cyan) and the lamins are cleaved and become cytoplasmic (as shown by the germline green becoming darker). The chromatin condenses and fragments as germline cell death progresses. The follicle cells then efferocytose the germline material (green vesicles) to clear it away.

During the early stages of oogenesis, three cell death events may occur—one for the entire germline cyst and two for the somatic cells [34,37,39]. The germline cyst cell death event takes place in region 2 of the germarium (Figure 3). This cell death is a response to a failure to pass a checkpoint that assesses the egg chamber’s structure and environment. If the egg chamber does not have the proper germline to FC ratio as can occur under starvation conditions, it will be directed to undergo a combination of apoptosis and autophagic cell death [65,66]. If the egg chamber has a proper structure and is being raised in a suitable environment, it will pass through the checkpoint to continue development [34,37,39,67].

The first form of somatic cell death takes place throughout early and mid-oogenesis [63]. From stages 2 through 8, apoptotic death has been detected in stalk cells. As they leave the germarium, egg chambers have too many stalk cells which, if they persist, have been shown to reduce fecundity. Egg chambers that lack stalk cells have been shown to merge with adjacent egg chambers. To achieve the optimal egg chamber production, stalk cells are overproduced early and die as development progresses [63].

The second form of somatic cell death event takes place prior to stage 5 (Figure 3) [34,37,39,67]. During the differentiation of the FC, polar cell, and stalk cell populations, 3–6 polar cells are produced at each pole. Prior to stage 5, this polar cell number must be adjusted to 2, and the excess polar cells are eliminated by apoptosis [64,68]. While the germarial checkpoint cell death occurs sporadically, extraneous polar cell death is necessary for the development of the anterior-posterior axis of the egg chamber [34,37,39,64,67,68].

### 3.1. Cell Death during Mid-Oogenesis

Once the proper number of polar cells and various FCs are designated at the end of stage 6, the egg chamber once again encounters a checkpoint [19,37,38]. During stages 7–9 of mid-stage oogenesis, the health of the egg chamber and the fly’s environment are assessed one final time. A defective egg chamber or a stressful environment containing harmful chemicals, cell phone radiation, predators, or even just lacking food can lead to the destruction of the egg chamber [32,35,69,70,71,72,73]. This final checkpoint occurs just prior to the energy-intensive production of yolk. If the egg chamber and environment fail to pass this checkpoint, the entire egg chamber is eliminated to avoid wasting the energy necessary to produce yolk for a fly embryo that is unlikely to survive (Figure 3) [32,33,34,37,38]. 

Although several environmental perturbations can induce cell death during mid-oogenesis, the best characterized stimulus is starvation [37,73]. Starvation-induced cell death has been categorized as primarily apoptotic due to the morphological characteristics and its requirement for the effector caspase Dcp-1 [32,74]. One noteworthy feature of starvation-induced egg chamber death is its synchronicity; rather than each NC degrading at its own rate, the entire germline undergoes the same processes simultaneously (Figure 3) [19,30]. To begin, all of the NC nuclei become disorganized and condensed. Concurrently, the lamina that line the nuclei degrade, thus dissociating from the chromatin and becoming cytoplasmic [75]. As death progresses, the NC nuclei continue to condense and fragment, while the rest of the germline material is cleared away [19,30,75]. 

Germline clearance is managed by the surrounding FC layer, the nonprofessional (and only) phagocytes of the ovary (Figure 3) [19,30]. As the germline dies, the FCs synchronously transition from their quiescent, support state to an active, phagocytic state. The FCs gradually increase in size as they efferocytose, or engulf, the entire germline. Once the germline material is cleared, the FCs, too, undergo cell death [19,30].

### 3.2. Engulfment Machinery

Much of the machinery that regulates engulfment was first identified in *C. elegans* [76,77,78]. There, two partially parallel pathways were identified, Cell Death Abnormality (CED)-1/-6/-7 and CED-2/-5/-12 which both signal to the GTPase CED-10 [79]. Each of these pathways has been evolutionarily conserved. In *Drosophila*, the pathways are Draper (Drpr)/Ced-6/Eato and Crk/Myoblast city/Ced-12, which both signal to the GTPases Rac1/2 [77,78,79,80,81,82,83]. During starvation-induced death in mid-oogenesis, both of these pathways are required in the FCs for proper clearance. When either *drpr* or *Ced-12* is knocked down in the FCs, the FC layer fails to clear away the entire germline [84].

*draper (drpr)*, the *ced-1* ortholog, plays several roles in the phagocytic process of the ovary [30,84]. To begin, Drpr acts as a key engulfment receptor that recognizes germline “eat me” signals and activates signaling in the FC. These signals trigger Rac1 to induce a conformational change and to activate the JNK signaling pathway. While the FCs start to engulf the cell corpse, the JNK signaling pathway leads to the expression of several genes, including *drpr*, which increase the efficiency of the phagocytic process [30]. As the clearance process continues, Drpr remains associated with the phagosomal membrane that surrounds the cell corpse, thus Drpr is also internalized by the FC [84]. Within the FC, Drpr interacts with the corpse processing machinery leading to the corpse’s eventual acidification [30,84]. 

*drpr* and the JNK signaling pathway are both required by the FCs for proper germline clearance [30,84]. If *drpr* expression is inhibited, engulfment is greatly reduced, vesicles containing corpses accumulate, and acidification fails to occur. Without *drpr* or JNK signaling, the engulfment process stalls, leaving the germline uncleared, while the FC layer dies [30,84].

*Drosophila* have two *ced-10* orthologs, *Rac1* and *Rac2*, that are both utilized during engulfment [83,85]. While knocking down *Rac1* and *Rac2* in the FCs of healthy, well-fed egg chambers does not produce a phenotype, in dying egg chambers each gene has a different effect on clearance. When *Rac1* is blocked, the FCs fail to engulf the germline material and die prematurely leaving dying germline behind. When *Rac2* is knocked down, some engulfment still occurs, but the FCs fail to enlarge as they take up the germline material inevitably blocking further engulfment. These findings suggest that while only *Rac1* is required for engulfment, both *Rac1* and *Rac2* are required to recycle material back to the cell membrane and cortex to increase the size of the FC cell and continue the process of engulfment [83]. 

### 3.3. Follicle Cell Genes Can Affect Germline Cell Death during Mid-Oogenesis

Although germline cell death is primarily apoptotic, the JNK signaling pathway, *drpr*, and several other genes expressed in the FC have also been found to play a non-cell autonomous role [19,30]. By inhibiting JNK activity in the FCs using a dominant negative version of *bsk*, it was demonstrated that the NC nuclei do not fragment completely, thus NC death is not completely autonomous. While *drpr* loss causes similar delays in the death of the starved germline, overexpressing *drpr* results in the death of otherwise healthy egg chambers. As the engulfment machinery of one cell is inducing the death of another cell, this form of cell death fits the definition of phagoptosis [19,30].

Additional evidence for the role of the FCs in promoting germline death comes from a recently published RNAi screen of the kinome that identified several genes that lead to an “undead” phenotype in which the germline remains intact, while the FC layer dies away [86]. The “undead” egg chamber phenotype was previously described in flies lacking the executioner caspase Dcp-1 or overexpressing the apoptosis inhibitor DIAP1 in the germline [30,74,87]. When any of the kinase genes *Taf1*, *ksr*, *Wnk*, *vari*, *CG7766*, *CG7156*, *Ask1*, *tkv*, *RIOK1*, or *SNF4Agamma* were knocked down in the FCs, the undead phenotype was observed, demonstrating that some signal from the FC must play a role in the death of the germline [86]. 

## 4. Non-Autonomous Developmental Death by Nonprofessional Phagocytes

**Figure 4:**
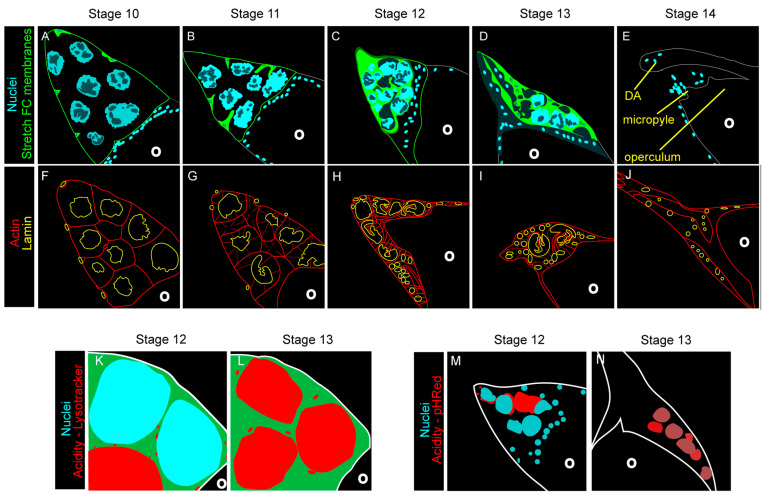
Developmental death of the nurse cells is orchestrated by the stretch follicle cells. (**A**–**E**) Images of the anterior portion of egg chambers during the late stages of oogenesis. (Adapted from [29]). (**A**) The anterior end of a stage 10 egg chamber with dispersed NC nuclei and surrounding FCs. (**B**) In a stage 11 egg chamber, NCs become smaller as they dump their cytoplasmic contents into the oocyte. Soon after, the stretch FCs invade the space between the NCs. (**C**) By stage 12, all of the cytoplasmic contents have been dumped into the oocyte and the stretch FCs have completely surrounded the NCs. (**D**) During stage 13, the NCs start to disappear and the dorsal appendages (DA) start to grow. (**E**) By stage 14, the NCs are completely eliminated, the DAs have fully extended, and the micropyle and operculum are complete. (**F**–**J**) Actin and lamin dynamics during developmental nurse cell death. (Adapted from [75]). (**F**) During stage 10, the only visible F-actin is cortical. The NC lamina, however, have already begun to fold creating crenellations (as compared to the smooth lamina of the stretch FCs). (**G**–**I**) During stages 11–13, actin bundles extend into the nucleus and the lamina undergoes additional folding, forming involutions and focal disruptions. (**J**) By stage 14, the only lamins present are those of the stretch FCs. The actin again can only be found subcortically. (**K**,**L**) NC nuclei become acidified during stages 12 and 13 (Adapted from [31]). (**K**) During stage 12, acidic organelles in the stretch FCs can be seen surrounding the NCs. (**L**) During stage 13, NCs become acidified. (**M**,**N**) NC acidity originates in the stretch FCs (Adapted from [31]). (**M**) During stage 12, acidity can be seen at the periphery of the nurse cells, near the stretch FCs. (**N**) During stage 13, acidity has flooded into the rest of the NC remnant. (**K**–**N**) The oocyte (O) is not affected by acidity.

At the end of oogenesis, a major cell death event occurs when the 15 NCs are eliminated prior to stage 14 (Figure 4A–E). A terminal stage 14 egg chamber is a large structure composed of an oocyte surrounded by the chorion and a layer of FCs [40,50,52]. At the anterior end of the egg chamber are three new structures, the micropyle which allows sperm to enter and fertilize the oocyte, the operculum which enables larval hatching, and the dorsal appendages which allows for oxygen exchange (Figure 4E) [58,59,88]. A pair of long, thick, fully formed dorsal appendages is the hallmark by which stage 14 egg chambers are identified. Upon looking at the stage 14 egg chamber, there is one striking omission, or rather 15, the NCs. To create this fully developed egg chamber, the NCs are eliminated in a developmentally programmed, phagocyte-dependent cell death [29,31,75,82]. To reduce confusion with mid-stage cell death in the ovary, this death will be referred to as developmental death.

### 4.1. Morphology of Developmental Cell Death

The beginning of the end for the NCs starts as soon as the FC layer remodeling is complete in stage 10 (Figure 4A) [29,38,75]. During stage 11, the NCs rapidly transfer their cytoplasmic contents through their ring canals to the oocyte in a process known as dumping. As the NCs continue dumping, the stretch FCs, which were in a holding pattern at the periphery of the egg chamber, invade the spaces between the NCs (Figure 4B). By the time the NCs are completely surrounded by the stretch FCs in stage 12, they have dumped as much of their cytoplasmic contents into the oocyte as possible (Figure 4C). The remaining NCs are not much more than plasma membranes with a nucleus. During stages 12 and 13, the NC remnants are removed such that by stage 14 the only germline remaining is the oocyte (Figure 4C–E) [29,38,75]. 

The morphology of developmental death and clearance of late oogenesis is strikingly different from the apoptosis and efferocytosis of mid-oogenesis. The most apparent difference between the two is the intimate relationship seen between the dying NCs and the stretch FCs [29,38,75]. Unlike mid-oogenesis death which takes place prior to any noticeable activity from the FCs, developmental death does not begin until stretch FCs begin to invade the NC space. Additionally, while the stretch FCs make contact with the NCs, there is no obvious engulfment taking place, the NCs just seem to vanish. Finally the dumping process is unique to developmental death [29,38,75]. In contrast, the entire germline, NCs and the oocyte, including the plasma membrane, cytoplasmic contents, and nuclei, is phagocytosed by the FCs during starvation-induced death of mid-oogenesis [30,32]. So, what precisely is happening to the NCs and what role do the stretch FCs play in their death? 

### 4.2. Stretch Follicle Cells Are Required for the Phagocyte-Dependent Developmental Cell Death of Nurse Cells

As apoptosis and autophagy play a clear role in starvation-induced death during mid-oogenesis [32,33,36,66], their impact on developmental death was investigated. Early studies demonstrated that NCs were acidified and contained activated caspases during stages 12 and 13 of oogenesis, thus suggesting that autophagy and apoptotic pathways were engaged during developmental death [87,89,90]. Further investigations used fly mutants to block either route, which led to a small number of persisting NC nuclei (PN) in the stage 14 egg chambers [87,91,92]. As PN are evidence that the NCs are not dying and/or being cleared away, this evidence supported the hypothesis that apoptosis and autophagy were key players in developmental death. In follow-up studies, however, the roles of autophagy and apoptosis in developmental death were diminished [29,93,94].

In one study, *Dcp-1*, *Diap1*, *Atg7*, and *Atg1* were used to investigate the role of apoptosis and autophagy in NC death [94]. Surprisingly, when both pathways were blocked, less than half of all stage 14 egg chambers contained any PN. Additionally, in egg chambers that contained PN, most only contained 1–3 PN, thus 12–14 nuclei were still cleared. Together, these data suggest that apoptosis and autophagy are not major participants in NC death and clearance [94]. 

In contrast to the mild phenotypes seen by inhibiting apoptosis and autophagy, striking effects on NC developmental death were observed when the stretch follicle cells were genetically manipulated [29]. In a key experiment, *Diap1* was knocked down in the stretch FCs to genetically ablate them. The resultant stage 14 egg chambers showed several defects including PN with unfragmented DNA and NCs that did not transfer their materials to the oocyte, a phenotype referred to as dumpless. These results showed that stretch FCs are required for developmental death [29], but how?

### 4.3. Molecular Mechanisms of Nonprofessional Phagocytes during Nurse Cell Death

Although developmental death is morphologically different from germline death in mid-oogenesis, some of the same genes play a role. *Ced-12* and *drpr* were found to be required in the stretch FCs for proper developmental death [29]. Loss of *drpr* or *Ced-12* expression resulted in, on average, more than 8 of the 15 NC nuclei persisting. When both genes were blocked, more than 11 PN were present, demonstrating that *drpr* and *Ced-12* play roles in two separate pathways [29].

The JNK pathway was also found to play a role in developmental death and clearance [29]. When JNK pathway components such as *kayak*, *jra*, and *bsk* were blocked in the stretch FCs, there were as many as 8 PN in stage 14 egg chambers. However, the regulation of the JNK pathway in late-stage oogenesis is different from that in mid-oogenesis. During mid-oogenesis, Drpr activates the JNK pathway early in the engulfment process [30]. As engulfment proceeds, the JNK pathway upregulates the expression of *drpr*. In late oogenesis, the JNK pathway still upregulates *drpr* expression, but Ced-12, not Drpr, was required for JNK activation [29] Thus, *drpr* and *Ced-12* have a complex relationship where they work in separate clearance pathways, but Ced-12 can also trigger the upregulation of *drpr.*

### 4.4. Collapse of the Nurse Cell Nucleus

Although most of these data indicate that FC genes play a role in clearance, several of these genes, specifically *drpr* and *Ced-12*, also regulate developmental death [29,75]. The first indication of NC death, the destruction of NC nuclei, begins in the interval between stages 10 and 11. Nuclear permeabilization was visualized by the transition of a nuclear stain to the cytoplasm indicating that nuclear membrane had begun to degrade. When *drpr* and *Ced-12* were knocked down in the stretch FCs, the transition of the dye was delayed in several nuclei. Additionally, the TUNEL staining that occurs during stages 12 and 13 is abolished when *drpr* is knocked down in FCs. Together, these data indicate that nuclear fragmentation is, in part, regulated by *drpr* and *Ced-12* [29]. 

The destruction of the NC nuclei during developmental death is structurally different from the germline death of mid-oogenesis [75]. During mid-oogenesis death, the nuclear lamina quickly degrades and the germline chromatin condenses and fragments synchronously (Figure 3). In developmental death, the nuclear lamina remains in contact with the chromatin until the bitter end. Additionally, the nuclei do not condense and fragment synchronously, rather they become compressed in an asynchronous fashion [75]. 

Nuclear compression is regulated by actin [52,75,95]. During stage 10, actin bundles form at the NC periphery and extend to the NC nuclei. As cytoplasmic dumping commences, the actin bundles push the NC nuclei up and away from the ring canals, thus preventing the formation of blockages [52]. As the actin continues to extend, it compresses the NC nuclei, forming gaps in the chromatin [75]. The structure of these gaps can be further visualized by examination of nuclear lamins (Figure 4F–J). Throughout developmental death, the nuclear lamina become increasingly folded resulting in the formation of crenellations at stage 10, then deeper involutions by stage 12, and finally the lamina becomes discontinuous and degrades in stage 13. Lamina degradation heralds the final destruction of the nucleus [75].

Stretch FCs are required for the maintenance of the actin bundles [75,95]. When stretch FCs are genetically ablated, the actin bundles still form during stage 10, but degrade by stage 12 [75]. The NC nuclei start to compress, but with the loss of the actin, they do not form as many involutions nor do they completely degrade. During a recent study, it was found that actin from stretch FCs extends towards the NCs in a similar time frame, from stages 11 to 12 [95]. It is possible that these FC actin extensions are there to stabilize the NC during the clearance process and, when the stretch FCs are ablated, there is no support system for the NC actin bundles. Without a support system, the actin bundles degrade and the NC nuclei persist [75,95].

As the NCs degrade, a series of calcium bursts can be detected within the stretch follicle cells [95]. Calcium bursts have also been seen in professional phagocytes such as macrophages [96,97,98]. During conventional phagocytosis, receptors are activated when they come into contact with an eat me signal [2]. Once activated, phagocytic receptors have been shown to induce calcium bursts downstream. Calcium bursts then prime the phagocyte by rearranging the actin cytoskeleton to prepare for engulfment [95,96,98]. Although the NCs are not cleared by engulfment, the actin dynamics seen in the stretch FCs could be regulated by calcium bursts once phagocytic receptors such as Drpr are triggered.

### 4.5. Acidification and Nurse Cell Destruction

As the egg chamber transitions to stage 13, the NC nuclei become acidified and finally degrade asynchronously (Figure 4K–N) [29,31,75]. In engulfment, acidification occurs when the mature phagosome merges with the lysosome [84]. Although NCs are not phagocytosed during developmental death, the acidification is still dependent on the surrounding FCs [29,31,75]. After genetically ablating the stretch follicle cells, acidification of the NC remnants is completely lost. Upon further investigation, it was found that *drpr* and *Ced-12* were required in the FCs for acidification of the NCs during developmental death and clearance [29].

Using LysoTracker, an acidophilic dye, and pHRed, a membrane-bound pH detector, the process of NC acidification was visualized [31]. Together, these indicators demonstrated that the acidity originated from regions of the NCs that came into contact with stretch FCs then spread to the entire NC remnant (Figure 4K–N). These results suggested that the acidity is transported from the stretch FCs to the NCs [31].

To identify the genes required in the FCs for this acidification process, several lysosomal genes were investigated [31]. Genetic perturbation of cathepsins and V-ATPase genes in FCs demonstrated several PN in stage 14 egg chambers. Cathepsins are proteases that require acidic conditions for activation. V-ATPases utilize ATP to generate an acidic gradient across a membrane. Thus, it can be hypothesized that V-ATPases generate the acidic environment where the cathepsins will work, but how do the FC lysosomes get to the nurse cells [31]?

One possibility is that lysosomes fuse with the stretch FC plasma membrane, thus V-ATPases can work at the interface of the stretch FCs and NCs, while the cathepsins are exocytosed [31]. Tagged V-ATPase proteins were found to localize at the plasma membrane of stretch FCs in regions adjacent to NCs. Oddly enough, lysosomal markers, such as LAMP1, were not found in the same location and when exocytosis genes, such as SNAREs, were knocked down, V-ATPases still localized to the plasma membrane, indicating that these V-ATPases were not a result of the fusion of lysosomes to the plasma membrane [31]. 

Additional evidence demonstrating that V-ATPases did not originate from the lysosomes came from the cathepsins, specifically CP1 [31]. CP1 presence in the NCs, like acidification, was non-autonomous; CP1 was built up in the stretch FCs then deposited in the NCs. Deposition of CP1 into the NCs required exocytosis genes and occurred after NC acidification by the V-ATPases Thus, lysosomal fusion with the plasma membrane could not be supplying both the V-ATPase-sourced acidification and CP1 as their presence would be simultaneous rather than sequential. Although V-ATPases are considered lysosomal, there are several instances in which they are found at the plasma membrane independent of the lysosomes, such as in osteoclasts and cancer cells, thus lysosomes may not play a role in this particular context [31]. 

Whether or not lysosomes play a role, acidification is one of the final steps required for NC destruction [29,31,75]. Once the NCs are acidified, CP1 is deposited in the nuclear remnants and becomes activated [31]. Activated CP1 then starts to degrade several substrates including lamins [75]. As the NC nuclei are stripped of their protective lamins, DNaseII degrades the chromatin [99]. 

Several questions remain regarding acidification of the NCs by stretch FCs. First, although V-ATPases still localized to the stretch FC plasma membranes in the absence of exocytosis genes, acidification of the NCs was significantly reduced, thus begging the questions what is being affected by the exocytosis genes and what other proteins are involved in acidification? Second, since exocytosis genes were required for cathepsin release into the NCs, but LAMP1 was not present at the plasma membrane, where are the cathepsins coming from? Third, the lysosomal gene *dor* is required for the breakdown of lamins and thus chromatin, so do lysosomes play a role in NC destruction? Finally, what is the connection between *drpr*, *Ced-12*, and acidification?

The connection between *drpr* and acidification may be through the V-ATPases [100]. V-ATPase expression is regulated, in part, by the transcription factor Mitf. When knocked down in the FCs, Mitf led to reduced acidification and increased PN in the NCs, thus Mitf is most likely regulating V-ATPases in the FCs. Mitf, in turn, must be tightly regulated as any change in Mitf regulation results in PN. Mitf expression has been shown to be modulated by TORC1 in other tissues [101]. TORC1 is a complex of proteins that, amongst other things, regulates autophagy by inhibiting Atg1 [102]. In the *Drosophila* brain, TORC1 activation has been found to rescue the corpse accumulation phenotype seen in *drpr* mutants [102]. This rescue implies that TORC1 may act downstream of *drpr* and could provide the link between *drpr* and acidification: *drpr* regulates the TORC1 complex, which regulates Mitf, which regulates V-ATPases which regulate acidification of the NCs.

### 4.6. Two Nurse Cell Nuclei “Egg” Ceptions

Although we theorize that developmental NC death is phagoptotic in nature, there are two exceptions [103]. During the production of the egg chamber, the germline precursor cell undergoes 4 rounds of mitosis to produce the oocyte and 15 NCs in a specific manner. Each of the resultant NC has a characteristic number of ring canals and is located at a certain distance from the oocyte. During stage 10B, just before dumping, one to three of the NCs located closest to the oocyte undergo a unique form of cell death in which the plasma membranes separating the oocyte and NC are briefly fused creating a large gap through which the NC nucleus exits the NC and enters into the oocyte [104]. Once the oocyte absorbs the NC nucleus, the plasma membranes reform leaving just a tiny gap between the oocyte and NC. Meanwhile, the NC nuclei break down quickly such that by stage 11 there is no evidence of the extra nuclei in the oocyte. Egg chambers that do not complete this process demonstrate abnormal DA morphologies and reduced viability [103].

Although the paper revealing this phenomenon briefly discussed the ramifications of this nuclear transfer, there are still so many questions. What other defects do oocytes without the extra nuclei suffer? What is the purpose of the two NC nuclei? What remains after the NC loses its nucleus and completes dumping? What genes and proteins regulate this process? Do the stretch follicle cells play a role?

### 4.7. Death and Clearance of the Follicle Cells

Once the FCs clear the NCs and deposit chorion on the exterior of the oocyte, their role in oocyte development is complete and they proceed to die. It has been proposed that FC death does not involve caspases and is strictly autophagic [104,105]. While the FCs die, the oocyte passes into the oviduct, thus removing the FC layer. The dying FCs are then phagocytosed by the epithelial cells of the lateral oviduct [104,105]. As most of this research was performed in other Dipteran species, this requires confirmation in *Drosophila melanogaster.*

## 5. Nonprofessional Phagocytes and Phagoptosis beyond the Ovary

While the *Drosophila* ovary provides us with many opportunities to learn about cell death, clearance and other functions, several other *Drosophila* organs use nonprofessional phagocytes. Two such organs are the testes and the brain. Testes, like ovaries, are relatively simple, repetitive structures that are straightforward for study. The brain, with its hundreds of thousands of cells distributed through several lobes, allows for investigations of more complex interactions. 

Like in the ovary, protein starvation can be used to investigate cell death and phagocytosis in the testis [106]. To conserve energy, the testes reduce the number of active germline stem cells and induce sperm cell precursors, called spermatogonia, to die early in spermatogenesis. Like in the female germline, spermatogonia death is regulated, in part, by their encapsulating cells [106]. These encapsulating cells, known as cyst cells, provide survival and differentiation signals to the spermatogonia. When starved of protein, the cyst cells undergo apoptosis which leads to an acidic, non-apoptotic death in the spermatogonia [106,107]. 

As the spermatogonia die, JNK signaling and the number of lysosomes increase within nearby surviving cyst cells [106]. JNK signaling in the cyst cell has been shown to be required for complete spermatogonia death. Interestingly, loss of this JNK signaling negatively affects germline stem cell survival. Taken together, these suggest that surviving cyst cells are nonprofessional phagocytes that promote the nonautonomous death and engulfment of the germline to provide energy necessary to maintain the germline stem cells [106]. Although there are many similarities to death in the ovary, starvation-induced death in the testes not only prevents energy waste, but recycles the germline material back to the stem cell, a process that has not been seen in the ovary. Thus, by researching other tissues, even very similar ones, new processes can be found.

While the ovary and testes have a single nonprofessional phagocyte population, the *Drosophila* brain has at least three—ensheathing glia, cortex glia, and astrocyte glia [19,108]. During the *Drosophila* lifecycle, there are several time points at which cells in the *Drosophila* brain die and are cleared [48,109]. These death events make it possible to study how cell death and clearance are regulated by other types of nonprofessional phagocytes. 

In addition to its many nonprofessional phagocytes, the brain has provided opportunities to study phagoptosis. A recent study demonstrated that phagoptosis could be induced in the brain when the phagocytic receptors Drpr or Simu were overexpressed in glia [27]. Such overexpression led to loss of dopaminergic and GABAergic neurons as well as impaired motor function and reduced life span. As many human genes have been evolutionarily conserved, such findings may be useful for understanding and treating neurodegenerative diseases such as Huntington’s disease and Alzheimer’s disease. 

## 6. Concluding Remarks

As it relies exclusively on nonprofessional phagocytes for clearance and its egg chambers undergo a variety of cell death events, the *Drosophila* ovary provides a versatile and optimal environment for studying nonprofessional phagocytosis. During starvation-induced death, the entire germline undergoes a synchronous, predominantly apoptotic death and is subsequently efferocytosed by the surrounding FCs. While there are several genes that have demonstrated non-autonomous roles, caspases are still critical for germline death and the FCs function as nonprofessional phagocytes.

**Figure 5:**
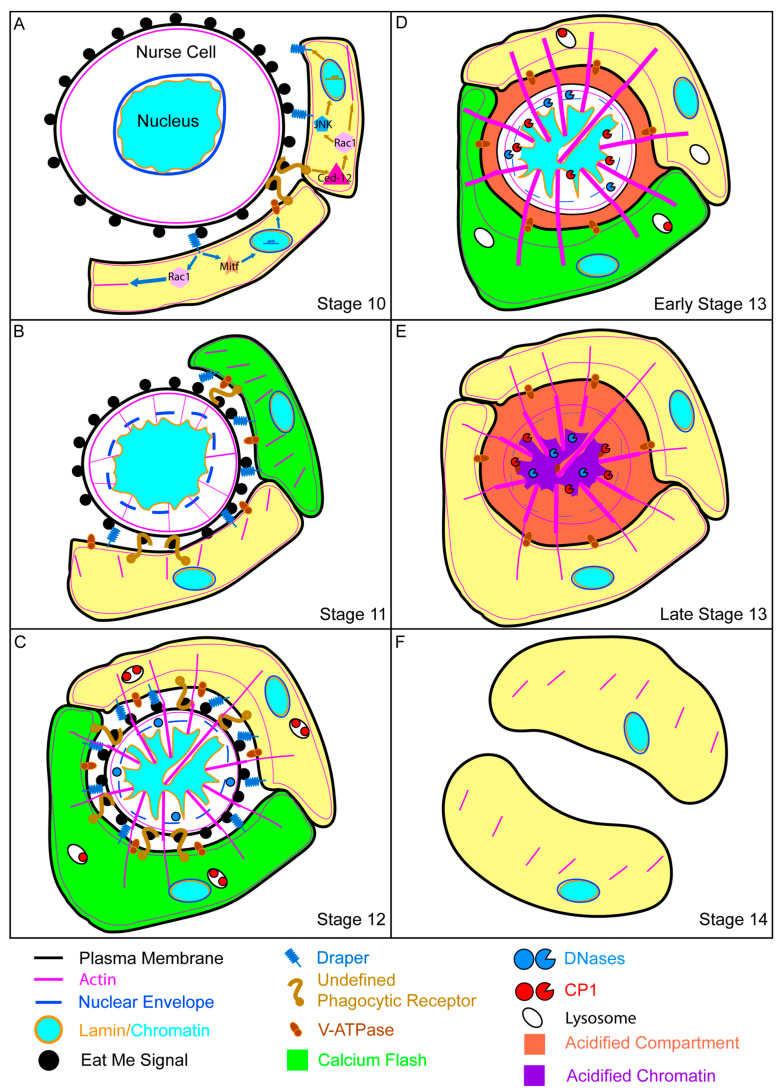
A model of developmental death and clearance of the nurse cells. (**A**) NCs of stage 10 egg chambers are at the cusp of death. The NC nucleus is already starting to fold, but the only visible actin is cortical. The NC has exposed a hypothetical eat me signal which is recognized by the phagocytic receptors on the FC membranes. When the *Ced-12* pathway is triggered, it likely signals through Rac1 to the JNK pathway to the nucleus to increase production of a variety of proteins including Drpr. Activation of the Drpr pathway may send signals to the nucleus via MITF to produce V-ATPases. Drpr and Ced-12 also signal Rac1 which regulates the conformational changes of the actin cytoskeleton. (Note: the processes and machinery seen early stages of developmental death and clearance are continuing throughout subsequent stages of developmental death). (**B**) The stage 11 NC can be seen to get smaller as it dumps its cytoplasm into the oocyte. The nuclear envelope has permeabilized and NC actin has begun to push into its nucleus. The stretch FCs have started to surround the NC and their actin is beginning to reach towards the NC. (**C**) By stage 12, the NC has dumped all of its cytoplasmic contents, leaving just a nucleus and some proteins surrounded by a membrane. Stretch FC actin is interacting with NC actin to support it as it pushes further into the nucleus forming involutions. The V-ATPases have started to acidify the NC compartment and inactive cathepsins are waiting in the stretch FCs. (**D**) The NC nucleus becomes acidified in early stage 13. Cathepsins enter the NC and begin to cleave lamins. DNaseII cleaves the NC chromatin. (**E**) Towards the end of stage 13, lamins are gone and the chromatin is being thoroughly degraded. (**F**) By stage 14, the NC has been eliminated and the stretch FC actin has receded. The spent phagocytic receptors have been endocytosed and the stretch FCs are preparing for their own end. The location of the NC material is still being elucidated.

As the cytoplasmic contents of the NCs are required for the complete development of the oocyte, it coheres that active caspases are not prevalent during developmental death and that the stretch FCs do not engulf the NCs. By carefully regulating the destruction of the NCs, the stretch FCs preserve the NC contents and allow them to be dumped into the oocyte. Only once the oocyte is fed are the NC remnants crushed, acidified, and degraded (Figure 5).

There are still many open-ended questions regarding developmental death in the *Drosophila* ovary. First, what signals the developmental death process to begin? Do the NCs present “find me” and “eat me” signals for the stretch FCs or do the stretch FCs just murder the NCs in due course? As all follicle cells are potential nonprofessional phagocytes, why are the stretch FCs, and not the main body FCs, phagocytic during late oogenesis? Where do the NC remnants go once they are dead? While neither apoptosis nor autophagy play a major role in NC death and degradation, some of their components are still present in late stage egg chambers; how do they interact with phagoptosis? Does *drpr* regulate acidification of the NCs through Mitf? Does *Rac2* play a role in developmental death? By answering these questions, we will have a further understanding of how nonprofessional phagocytes eliminate their prey.

Nonprofessional phagocytes, like the follicle cells of the *Drosophila* ovary, provide an opportunity to study cellular machinery being used in atypical ways. The stretch FCs, for instance, utilize V-ATPases, which are commonly found in the lysosome, to acidify their prey extracellularly [31]. While such behavior is uncommon, there are several cell types that use similar methods including cells in the ear, epididymis, and kidney [110]. Additionally, the ABCA1 transporter, known as EATO in *Drosophila*, is typically utilized for lipid efflux [111]. In contrast, it has been proposed that EATO is used to recycle lipids to the membrane, thus allowing the membrane to grow and expand as it engulfs [82]. As ABCA1 is critical in diseases such as atherosclerosis and Tangier disease, studying its functions in other models may provide new insight [112,113]. Thus, nonprofessional phagocytes of the *Drosophila* ovary provide new contexts for exploring homeostatic processes.

Nonprofessional phagocytes are diverse and ubiquitous [2]. Throughout the body, several types of epithelial cells, including mammary epithelium and retinal pigment epithelium, become phagocytic when they interact with dying cells [16,20,21,114]. Interestingly, a recent study demonstrated that cells in the heart, kidney, and other viable tissues are also capable of homotypic efferocytosis indicating that many cell types can act as nonprofessional phagocytes [115]. By learning about death and clearance using a simple tissue such as the *Drosophila* ovary, findings could be extrapolated to predict those processes in more complex tissues.
